# Interventions to reduce the negative effects of alcohol consumption in older adults: a systematic review

**DOI:** 10.1186/s12889-018-5199-x

**Published:** 2018-03-01

**Authors:** Roxanne Armstrong-Moore, Catherine Haighton, Nicola Davinson, Jonathan Ling

**Affiliations:** 1 School of Nursing and Health Sciences, University of Sunderland, Sunderland, SR1 3SD UK; 2 Department of Social Work, Education and Community Wellbeing, Northumbria University, Newcastle upon Tyne, NE7 7XA UK; 3 Institute of Health and Society, Newcastle University, Newcastle upon Tyne, NE2 4AX UK; 4 School of Psychology, University of Sunderland, Sunderland, SR1 3QR UK

**Keywords:** Alcohol, Public health, Systematic review, Older adults

## Abstract

**Background:**

Older individuals are consuming alcohol more frequently yet there is limited evidence on the effectiveness of current interventions. This systematic review aims to investigate interventions that target alcohol use in individuals aged 55 + .

**Methods:**

CINAHL, ERIC, MEDLINE, Science Direct, PsychInfo, SCOPUS, Web of Science and socINDEX were searched using terms devised from the PICO (Population, Intervention, Comparison and Outcome) tool. Studies using pharmaceutical interventions, or those that investigated comorbidities or the use of other substances were excluded. Peer reviewed empirical studies written in the English language that compared the outcomes of alcohol related interventions to standard care were included in this review. Studies were appraised and assessed for quality using the relevant Critical Appraisal Skills Programme checklist.

**Results:**

Seven papers were included in this review. Six were conducted in the United States of America and one in Denmark. The interventions were carried out in primary care centres and in community based groups. The studies included in this review showed varying levels of success. Participants showed improvements in at least one area of alcohol consumption or frequency of consumption however, these did not always reach significance.

**Conclusion:**

Individuals in this age group appear to respond well to interventions aimed at reducing alcohol consumption. However, included studies had limitations, in particular many did not include a clear intervention description; leaving us unable to fully investigate the components required for success. Further research is needed on the effective components of alcohol interventions targeting older people.

## Background

Worldwide, alcohol-related diseases are responsible for 2.5 million deaths per year [1]. Therefore, alcohol consumption and associated negative impacts are a significant problem to public health. Projections from the World Health Organization (WHO) indicate that by 2025, alcohol consumption is expected to increase in almost half of the member states, something that will only be reversible with the implementation of “effective policy responses” [2]. The damage from alcohol use inflicts social and economic damage across many societies and this worldwide burden will increase if policy is not improved [2]. According to the WHO the highest levels of alcohol consumption are in the developed world, in particular Europe and the Americas, with intermediate levels of consumption in the Pacific and African regions with the lowest consumption in South-East Asia and the Eastern Mediterranean [2].

By 2050, around 22% of the world population will be aged 60 and over, with a significant proportion of these older individuals having a “pattern or level of drinking which places them at harm” p.656 [3]. In comparison to younger people, older adults are more susceptible to the detrimental effects of alcohol, as their tolerance to alcohol lowers with age. In addition, older people are also more likely to take prescription medications which, when taken with alcohol, can reduce effectiveness of medication, exacerbate side effects or even lead to the development of new illnesses [4].

Drinking more than five standard drinks per week and a history of an alcohol problem in men over the age of 50 has been found to quadruple the risk of developing psychiatric problems including depression and memory loss [5, 6]. Cognitive impairment can lead to an increased likelihood of falls [7] and because older people often have weaker bones, this can lead to hip fractures, which is one of the highest causes of death in the older population [8].

Significant events experienced during the life course have been associated with increased alcohol consumption. One such event is retirement which is associated with changes in drinking patterns [9]. However, the relationship between retirement and alcohol consumption is unclear. In their review of the literature, Zantinge et al. [10] found some studies reported an increase in alcohol use after retirement, while others reported a decrease, or no change. They concluded that individuals who retired involuntarily were generally more likely to increase alcohol consumption, with those choosing to leave less likely to change drinking pattern. There is also some evidence suggesting a reverse causality in this relationship between alcohol use and retirement, highlighting that men with existing alcohol problems who are eligible to retire are more likely to do so than men without such problems [11].

A recent review by Bhatia et al., [12] addressed recent advances in treatments for older people and the effectiveness of interventions for substance use problems as a whole. They concluded that older people are good at utilising interventions in this area and that they show positive outcomes. They do however note that the evidence base needs to be developed and refined. The current review is the first study to focus specifically on alcohol use in later life, as opposed to the broader topic of substance misuse.

The current study focuses on research that has evaluated alcohol interventions in older people. As alcohol is a complex problem, specific knowledge is needed to enable the development of effective alcohol interventions specifically for older alcohol users. Therefore, the aim of this study was to conduct a systematic review of interventions to reduce alcohol targeted at older individuals in order to examine the factors, conditions and motivations that contribute to successful intervention in this area.

## Method

### Design

A systematic review was conducted according to the Cochrane Handbook for Systematic Reviews of Interventions, which offers guidance on how to conduct reviews on healthcare interventions. The handbook was used to guide authors on planning the review, searching for material to include, assessing risk of bias and reporting results [13]. This systematic review is reported according to PRISMA [14] guidelines.

### Data Sources

Eight electronic databases (CINAHL, ERIC, MEDLINE, Science Direct, PsychINFO, SCOPUS, Web of Science and socINDEX) were searched by RA and checked by JL using the search terms outlined below (see Table 1 for search strategy for each database). The search was conducted in October 2017. In addition, the reference lists of included studies were subsequently hand searched in order to identify any other studies that would potentially be suitable for inclusion.

**Table 1:** Search strategy table

Database	PSYCHINFO/SCOPUS/Science Direct
Date	31/10/2017
Strategy	#1 and #2 and #3 NOT #4 NOT #5
#1	older OR older adults OR seniors OR geriatrics OR ageing OR aging
#2	alcohol or drinking or alcohol consumption
#3	intervention OR strategies
#4	pharmaceutical or child or young adult or teenage or adolescent
#5	secondary analysis
Limiters	Language – English, Published between 01/01/1990–31/10/2017
Database	Web of Science/Medline/SocIndex/CINAHL/ERIC
Date	31/10/2017
Strategy	#1 and #2 and #3 NOT #4 NOT #5
#1	TITLE: older OR older adults OR seniors OR geriatrics OR ageing OR aging
#2	TITLE: alcohol or drinking or alcohol consumption
#3	TOPIC: intervention OR strategies
#4	TOPIC: pharmaceutical or child or young adult or teenage or adolescent
#5	TOPIC: secondary analysis
Limiters	Language – English, Published between 01/01/1990–31/10/2017

### Search terms

Search terms were devised using the PICO tool, which aims to address the population, intervention, comparator intervention and outcome measures of a study [15]. The search terms used were as follows:

- Age – synonyms for older included older adults, seniors, geriatrics or ageing/aging
- Alcohol – synonyms included drinking, alcohol consumption, substance
- Intervention – synonyms included strategies.

### Search strategy

#### Study inclusion criteria

Any type of peer reviewed empirical study, written in English. Participants aged ≥55 years old and interventions that compared alcohol-related outcomes against a control group were included in this review. Definitions vary on what constitutes an “older” person. For this review, we focus on those aged 55 years old and above, unless stated otherwise. This age was chosen to allow as much data as possible, from retirement age through to elderly individuals. Studies had to have been carried out within the last 27 years, in order to understand the current stance of the literature and to provide a synthesis on interventions from this point. Literature was eligible for inclusion if it was published between 1990 and 2017.

#### Exclusion criteria

Studies that used pharmaceutical interventions, or those that investigated specifically comorbidities or use of other substances were excluded from this review. Studies that aimed to speculate outcomes for future interventions or evaluations of previous research and reviews were also excluded, as these did not bring any novel findings or information relevant to the research question posed. Articles that were not peer reviewed and empirical studies were excluded, as were studies not written in the English language, as translation services were unavailable.

#### Data extraction

Titles and abstracts of articles were assessed for eligibility based on inclusion/exclusion criteria. Full texts of all relevant articles were obtained and assessed for eligibility by RA and then checked independently by JL. Data were extracted from all eligible articles using a standard data extraction form and assessed for quality using the relevant Critical Appraisal Skills Programme checklist [16] studies were also checked for risk of bias, by checking results and funders. Authors were contacted where necessary to obtain and confirm data.

## Results

**Fig. 1:**
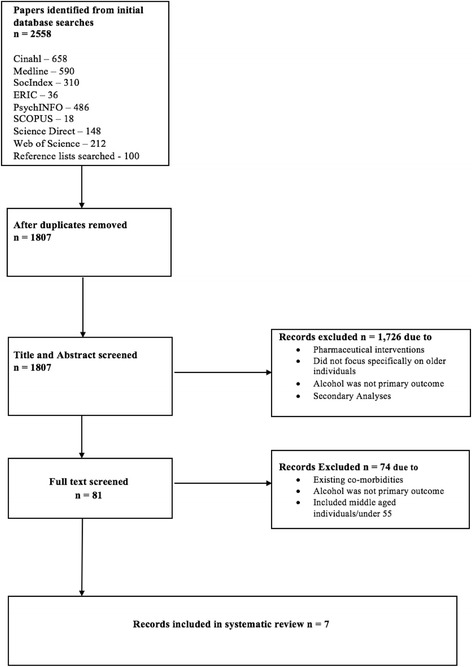
PRISMA flow diagram of included reviews

Figure 1 (Appendix 1) contains a PRISMA flow diagram of included reviews and shows the number of articles obtained at each stage of the review process. Table 2 shows the seven trials included in this review. A narrative synthesis is provided on the characteristics of the included studies, including outcome measures and critical appraisal of the interventions. As all interventions had different outcome measures it was not appropriate to conduct a meta-analysis. All included studies were randomised controlled trials; therefore the CASP Tool for Randomised Controlled Trials was used as the appraisal tool [16]. All trials in this study were considered to have appropriately met the validity and quality appraisal in terms of CASP.

**Table 2:** Details of Included Interventions

Study ID, country of origin and setting	Details of Sample	Allocation of Participants	Methods	Details of Intervention(s)	Mode of Delivery	Interventionist	Assessment periods	Outcome Measures and findings
Gordon et al. (2003) USA Primary care offices	*n* = 45 Age > 66 years old Convenience sample gained from waiting rooms of primary care centres. All were hazardous drinkers Randomly allocated to one of three groups: Motivational Enhancement (ME) *n* = 18 Brief Advice (BA) *n* = 12 Control group - care as usual (SC) *n* = 12	Allocated randomly to receive one of two interventions, or standard care as usual.	Enrolled and completed questionnaires at baseline.	ME – Included feedback, goal setting and consequences. First session lasted around 60 min, with two booster sessions of around 10–15 min. BA – one 10–15 min sessions focussing on advice Control group (usual care)- were given care as usual, no discouragement against talking about alcohol.	Face to face	Trained research interventionists	Baseline, 1, 3, 6, 9 and 12 months	Time Line Follow Back (TLFB) questionnaire used measure quantity and frequency of alcohol consumption. Also includes a tool to measure amount of drinks consumed in the last month, amongst other frequency measures. All measures included were self-reports. Each group showed decreases in alcohol consumption measures over time, however these were not statistically different to those who received standard care.
Hansen et al. (2011) Denmark No information on specific setting of intervention.	*n* = 772 Age > 50 years old Used AUDIT tool to identify only those who were heavy drinkers. Intervention Group *n* = 391 (*n* = 316 at 12 months) Control Group *n* = 381 (*n* = 300 at 12 months)	No information given as to how participants were randomised into groups	Information gathered on alcohol use at baseline.	BMI – Consisted of a conversation based on the principles of MI, designed to motivate individuals to change behaviour through open ended questions. Also were given an information sheet with information about local alcohol treatment and a brief telephone booster 4 weeks later. Control group received same leaflets about alcohol and local treatment. A pure control group was not included.	Face to Face, telephone booster	Nurses and Postgraduate Students	Baseline, 6 and 12 months.	Outcome measure was drinks per week. This particular study did not find any significant intervention effect on drinks per week.
Kuerbis et al. (2015) USA Primary care clinics	*n* = 86 Used CARET to ensure only selected at risk drinkers. > 50 years, mean age of just under 65 years old Intervention group *n* = 44 Control group *n* = 42	Participants were randomly assigned to the intervention or control group.	Participants completed assessment tool at baseline.	Intervention Group – received personalised mailed feedback outlining the risks specific to their alcohol use. Also received the *NIH Rethinking Drinking: Alcohol and Your Health* booklet Control group – did not receive anything	Mailed to Participants	n/a	Baseline and 3 months	CARET was used to measure alcohol risk score at 3 months. Significant reductions were found in the intervention group for binge drinking, alcohol use with a medical or psychiatric condition and alcohol with symptoms of a medical or psychiatric condition. Intervention groups were 72% less likely to be an at risk binge drinker and 92% less likely to be at risk due to a medical or psychiatric condition.
Fleming et al. (1999) USA Community based primary care practices	*n* = 146 for full 12 months Age > 65 years old Used Health Screening Survey (HSS) to include only those who were problem drinkers Intervention group *n* = 87 Control group (usual care) *n* = 71	Participants were randomly allocated to receive either the brief intervention (BI) or care as usual.	Participants completed assessment tool at baseline.	BI group – received booklet on general health and were also scheduled to see their personal physicians. Used BI protocol including a workbook containing feedback on individual’s behaviours and other educational resources. Had 2 × 15 min appointments, one month apart consisting of the intervention and then a reinforcement session. Control group (usual care) – only received a general health booklet.	Face to Face and telephone	Physician trained in internal or family medicine	Baseline, 3, 6 and 12 months. Family members contacted at 12 months for back up on results self-reports	Used drinks per week, levels of binge drinking and excessive alcohol use. The intervention group maintained lower levels of drinking throughout and reduced their weekly alcohol use. They also self-reported reduced amounts of binge drinking and excessive levels of drinking. These were statistically significant.
Ettner et al. (2013) USA Community based practice, 7 clinics	n = 1186 Age > 60 years old Used CARET to ensure only selected at risk drinkers. Intervention group *n* = 546 Control group (usual care) *n* = 640	Patients were randomly assigned to a group dependant on which group the physician they saw was assigned to.	Participants completed CARET and Alcohol- Related Problems Survey at baseline to determine risk and frequency of alcohol use, whether they had discussed their alcohol use with their physician and self-reported health care use.	Intervention group – used project SHARE which included personalised reports, education material, telephone counselling and physician advice. Control group (usual care) – received care as usual. Alcohol discussions were not discouraged	Face to Face and telephone	Physician for intervention and health educator for telephone counselling.	Baseline, 3, 6 and 12 months.	Alcohol- Related Problems Survey, CARET Primary outcome measure - Whether individual continued to be at risk. Secondary outcome measure – score on CARET, drinks per week, amount of discussions with physician and use of health care including trips to emergency care etc. All measures were self-reports Greater declines and fewer at risk statistically in intervention group compared to control.
Moore et al. (2011) USA Primary care sites	*n* = 631 Age > 55 years old All participants were at risk, as identified by the CARET tool. Intervention group *n* = 310 Control group *n* = 321	Participants were randomly assigned to either the intervention group or the control group.	Patients were at risk of alcohol misuse as determined by CARET at baseline.	Intervention group = received a multi-faceted intervention that included a personalised report, a booklet on alcohol and ageing, a diary to log levels of drinking, advice and telephone counselling Control group = only received a booklet on healthy behaviours	Face to face and telephone	Research assistant and primary care provider	Baseline, 3, 12 months	CARET, number of drinks consumed in the past 7 days and heavy drinking in the past 7 days. All measures were self-reports. 3 At 12 months, intervention group did not have lower levels of at risk drinking, however did reduce levels of alcohol consumption. Results were statistically significant, but researchers suggest that they may not provide clinical significance.
Fink et al. (2005) USA Community primary care practices	*n* = 665 Age > 65 years old Participants were eligible to take part if they had consumed more than one alcoholic drink in the last three months. Split into three groups, two experimental and one control. Experimental 1 (both physicians and patients received reports) *n* = 212 Experimental 2 (only patients received reports, physicians did not) *n* = 245 Control group (usual care) *n* = 208	Participants were randomly assigned to intervention or control groups.	Participants took part in either one of three groups, two being experimental and one being control.	Experimental group 1 – physician and patients received reports on the patients’ alcohol use, risks and problems. They also received personalised educational tools. Experimental group 2 – Only the patients received their reports, the physicians did not. They also received the personalised educational tools. Control group (usual care) – were not informed on their individual risks, nor did they receive any educational tools	Computerised report	Physician	Baseline and 12 months	Found reductions in hazardous drinking, reductions in harmful drinking and maintenance of non-hazardous drinking in both experimental groups when measured with CARPS – baseline, 12 months later. Measures were self-reports. Both experimental groups had lower risk drinking compared to control. Patient only reports led to reduced harmful drinking and less hazardous drinking. Combined reports only decreased total consumption.

This analysis of seven published randomised controlled trials, involved a combined total of 3531 participants. Six of the trials were conducted in the United States of America and one in Denmark. Studies were carried out in primary care centres and community-based groups or provided elsewhere [17–22]; the exact location is not specific in Hansen et al. [23]. The included studies showed that there were varying levels of success, all interventions showed improvements in at least one area of alcohol consumption or frequency of consumptions. This was not always significantly more than control groups and the potential reasons for this are explored below.

### Mode of delivery

All but one of the interventions were carried out face to face, with five of seven using telephone follow-ups. Some of the Included interventions were carried out solely by physicians (*n* = 2) [18, 22] whereas other intervention providers used a combination of either physician and health educators [17] (*n* = 1) or trained research assistants/interventionists, care providers or health educators or postgraduate students (*n* = 4) [19, 23].

### Type of interventions

Interventions used in trials included varying techniques and some used more than one intervention group, and these are listed below:

- Motivational Enhancement [19]
- Brief Motivational Intervention [23]
- Brief Advice [19]
- SHARE (Senior Health and Alcohol Risk Education) [17]
- Brief Intervention (BI) - used BI protocol including a workbook containing feedback on individuals’ behaviours and other educational resources. Two appointments, one month apart consisting of the intervention and then a reinforcement session [22]
- Personalised reports on risks and problems [18, 20]
- Educational tools [18, 21, 22]
- Diaries [20]
- Telephone Counselling [17, 20]

Control groups received either care as usual or booklets on alcohol or healthy behaviours. There were no apparent restrictions or discouragement from talking about alcohol in the control groups.

### Assessment periods

All interventions included in this study measured results across 12 months or less. Assessment periods varied, testing outcomes at either 1, 3, 6, 9 or 12 months and some focussed on more than one of these periods. Fleming [22] also contacted family members at 12 months for clarification of participants’ progress.

### Screening

Participants in the interventions were identified before the studies took part as heavy drinkers or hazardous/problem drinkers, apart from one study [18], which used participants who were eligible if they had consumed more than one alcoholic drink in the last three months. The participants included in 6 of the studies were not dependent drinkers. Hansen et al. state that dependent drinkers could be included in their study and were not excluded, however there is limited further detail on this [23]. The levels of hazard were determined using tools such as the Comorbidity Alcohol Risk Evaluation Tool (CARET) [17], Alcohol Use Disorder Identification Test (AUDIT) [23] Time Line Follow Back (TLFB) [19] and the Heath Screening Survey (HSS) [22].

### Outcome measures

The main outcome measure was reduction in reported alcohol consumption, as this was measured differently across studies, a meta-analysis was not appropriate. Other outcomes included riskiness of drinking, general health and education and knowledge in relation to alcohol. The specific outcomes achieved were considered and the tools used such as questionnaires and scales to determine frequency of alcohol consumption and health.

### Potential risk of bias

Studies were appraised for risk of bias, confirming that most of the included studies were funded either wholly or partially by the National Institute of Alcohol Abuse and Alcoholism. The study conducted by Hansen et al., was funded by the National Board of Health, Denmark [23]. There were no conflicts of interest declared in the papers, and none were funded by alcohol manufacturers, which has been suggested as a factor that can potentially jeopardise the integrity of conducted research [24]. A risk of bias table (Table 3) has been included in line The Cochrane Collaboration’s tool for assessing risk of bias in randomised trials [25].

**Table 3:**
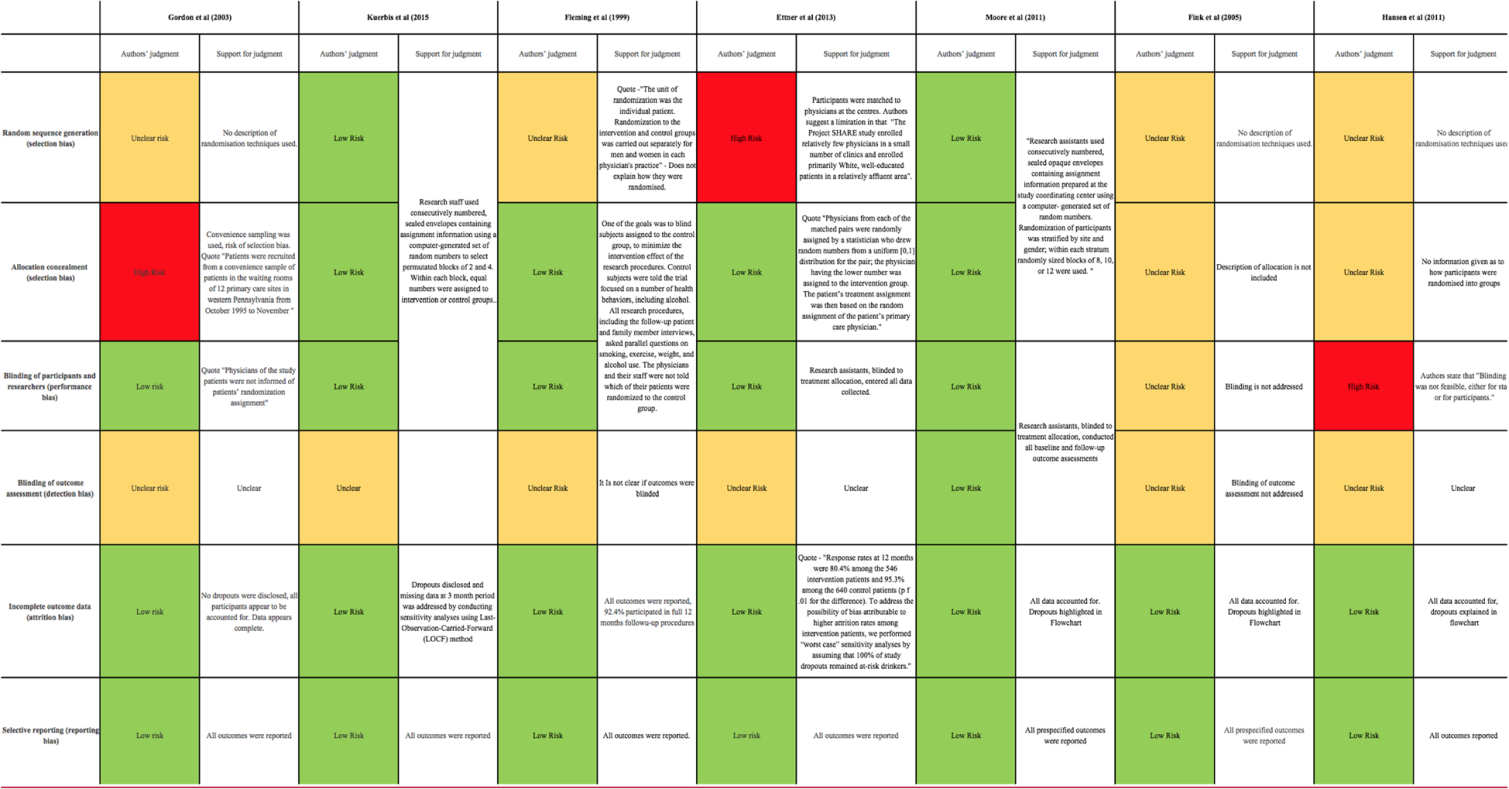
Risk of Bias table

Gordon et al. [19] used a relatively small sample of hazardous drinkers (*n* = 45) over the age of 66 years old, of which 87% were male and 69% were high school graduates. This study used the Time Line Follow Back [26] survey to measure frequency and consumption of alcohol consumption. The authors state that “multivariate tests” revealed significant reductions in alcohol frequency measures across all groups, regardless of intervention given over time - however this also included standard care. On inspection of the results, none of the delivered interventions showed a significant reduction when compared to the group who received standard care. This indicates that for older adults, merely raising the issue of alcohol consumption may be enough to get them to reduce their drinking. Gordon et al. suggest further research on the efficiency, cost effectiveness and patient preferences for future intervention development.

Hansen et al. [23] included 772 participants over the age of 50 who were heavy drinkers, as determined by the AUDIT tool. Males made up 49% of participants in the intervention group and 54% of the control group. Around 40% of this sample had spent 15 years or longer in education. Hansen et al. [23] found that alcohol consumption was reduced at both 6 and 12 months, however there were no significant differences between those who had the brief motivational intervention and the group.

Kuerbis et al. [9] tested the efficacy of a mailed screening and brief feedback intervention to reduce at risk drinking in 86 adults aged 50 and over. As discussed, this review was primarily investigating individuals over 55, however this study and the study by Hansen et al. was deemed appropriate for inclusion, due to mean ages of around 65 years old. At risk drinking was measured using the CARET tool. Of the 86 participants, 66% were male and 77% were educated to college level or higher; 88% of participants were non-Hispanic white. At 3 months, there were no significant differences in drinks per week between the two groups, although the CARET score did reduce in the intervention group (*p* < 0.01). However, this particular study was a pilot to test the efficacy and feasibility of the intervention, and thus included only a small number of participants.

Ettner et al. [17] investigated 1186 participants over the age of 60 years old who were at-risk drinkers measured using the Comorbidity Alcohol Risk Evaluation Tool (CARET; [27]), 1049 of whom completed the full 12 months of the Project SHARE intervention. Of the 1186 participants, 65.7% were male and 96.8% had attended at least high school. The authors concluded that the intervention was effective, at both 6 and 12 months, at significantly reducing alcohol consumption (*p* < 0.01), heavy episodic drinking (*p* < 0.01) and reducing patients’ visits to physicians and emergency departments (*p* < 0.01).

Fleming et al. [22] used a modified version of the Health Screening Survey (HSS) [28, 29] to include 158 participants over the age of 65 who were problem drinkers. Individuals were eligible to take part if they were men drinking more than 11 drinks per week (132 g of alcohol) or women consuming more than 8 drinks per week (96 g of alcohol), had 2 or more positive responses to the CAGE questionnaire, or had consumed 4 or more drinks per occasion for men or 3 or more drinks per occasion for women on 2 or more occasions in the last 3 months which was defined as binge drinking. Of this sample, 66.4% were male and 33.6% were female. The whole study population were described as well educated, with “higher proportions” of individuals being educated beyond high school. Individuals who received physician interventions significantly reduced their 7-day alcohol use (*p* < 0.01), episodes of binge drinking (*p* < 0.05) and frequency of excessive (more than 30 drinks per week for males and more than 13 for females) drinking (*p* < 0.05).

Moore et al. [20] investigated the effects of an intervention with multiple components (including a personalised report, a booklet on alcohol and ageing, a diary to log levels of drinking, advice and telephone counselling) on 631 participants over the age of 55. Participants were defined as at risk using the Comorbidity Alcohol Risk Evaluation Tool (CARET; [27]). This sample included 71% males, and 77% were educated beyond high school. They found the intervention at 3 months reduced the proportion of at risk drinkers (*p* < 0.01); participants were more likely to report having fewer drinks in the last 7 days (*p* < 0.001) and had a lower risk score (*p* < 0.01). However, at 12 months, only the group difference in the number of drinks consumed in the past 7 days remained significant (*p* < 0.05).

Fink et al. [18] carried out their intervention on 665 at risk participants over the age of 55, of which 47% male and 91% with high school education or higher. Risk and alcohol related problems were measured using the Computerised Alcohol Related Problems Survey (CARPS; [30]). Both intervention groups were associated with greater odds of lower risk drinking at 12 months follow up (both *p* < 0.05). This included the intervention group where only patients received their report on alcohol use and risks and the combination group where both physician and patient received the report. Linear regression showed that the combined report group had significantly decreased quantity and frequency of consumption compared to usual care (*p* < 0.05), however using only the patient report did not significantly decrease frequency of consumption. Both interventions led to a decrease in harmful and hazardous drinking compared to controls (*p* < 0.05).

## Discussion

This is the first systematic review that focuses solely on the effectiveness of interventions to reduce alcohol use in older individuals and in particular individuals aged over 55. There were improvements in frequency and amount of alcohol consumption in at least one area in all included interventions. Some interventions were successful, apart from Hansen et al., [23] and Gordon et al., [19] who found that whilst interventions show significant reductions over time, they did not show significant differences in comparison to the control group individuals who received standard care. The improvements included lower amounts of frequency and consumption [17–19] and reduced 7-day alcohol use ([20, 22]). In addition, other studies found lower rates of heavy episodic drinking and visits to physicians and emergency departments [17] a lower frequency of “binge drinking” episodes and frequency of excessive drinking [22] and harmful and hazardous drinking [18].

In a previous systematic review of interventions focusing on the wider issue of substance misuse in older adults, older adults were found to respond well to psychological treatments [12] Bhatia et al. found that although there were promising responses from participants in regards to current treatments; further examination should focus on a wider range of interventions which could be offered and may better suit older individuals. The current review begins to investigate these strengths of interventions, evidencing that individuals do appear to respond to psychological based treatments such as counselling and advice on behaviours, they also respond as well to educational tools, personalised reports that indicate their own level of risk and the use of diaries.

A common theme in this review was that studies often lacked a clear intervention description, which meant that determining which components were effective was problematic. The effective components were psychological based treatments such as counseling and advice on behaviours, educational tools, personalised risk reports and diaries. However, the information provided on these tools was not sufficiently detailed to clarify which specific elements of the interventions were effective. Information provided on the control groups that received “care as usual” was limited. One study provided some alcohol-specific information to control participants and some others (*n* = 3) provided a general health information sheet to participants; others explicitly stated that discussions surrounding alcohol were not discouraged (*n* = 3), however more information on the control groups would have been useful in assessing the effectiveness of the interventions.

Hansen et al. [23] found no significant difference between their brief motivational intervention group and the control group and they give numerous possible reasons for this. They stated that they included no pure control group for ethical reasons and that whilst control participants did not receive the brief motivational intervention, they were given leaflets on alcohol and treatment. This information alone may have resulted in the documented reductions as they concede that even the act of taking part in a health-based intervention may have led to a reduction in alcohol use. They also suggest that reductions could be due to “regression to the mean, social desirability bias and historical changes in alcohol consumption” p. 30.

All studies included were assessed as per the CASP guidelines. There are 11 questions included in the guidelines that help the researcher to assess trials systematically. By using this set of guidelines, this systematic review assessed each trial appropriately. All included trials were assessed and deemed to be valid.

Six of the seven studies included used some form of blinding outcomes, which adds to the validity of results. However Hansen et al. [23] did not use any blinding in their groups, declaring it unfeasible and Fink et al., [18] provide limited information on blinding. Fink et al. also admit that physicians in their three groups may have discussed the process, however they have no evidence that this did occur; this is a weakness of both included studies.

In terms of sample size, Gordon et al., [19] had a relatively small sample size, with no power calculation provided and the authors comment on this small sample as a potential limitation. Kuerbis et al. [9] also had a small sample for their pilot study. Sample sizes were larger in Ettner et al. (*n* = 1186) [17] however power was not addressed in this study, nor by either Fleming et al. [22] or Fink et al. [18] . Moore et al. [20] and Hansen et al. [23] addressed power, stating that numbers used ensured sufficient power, Hansen stating that power was sufficient providing that 75% of participants completed the full study, which they did. Using the support from other individuals, including family members and friends could be effective in reducing alcohol consumption. Only one study in this review used the support of other individuals within the intervention setting which consisted of speaking to family members about participants’ results [22]. Whilst this is useful to gain clarification on self-reported results, they do not explain how they affect alcohol-related behaviours.

Support from individuals could span beyond close friends and family members. Evidence suggests that social networks and groups to which an individual subscribes could also link to drinking behaviours and could include where an individual works or their age group. Views and behaviours in relation to drinking are linked to social background and may respond to interventions and public health messages accordingly [31]. Future research could investigate how family and social networks surrounding older individuals could contribute to a successful intervention.

All the papers included in this review contain at least some element of self-reporting. This may lead to socially desirable responding from participants, who do not want to disclose the full extent of their problems and under-report usage [32]. This can threaten the validity of trials and underestimate harm. Gordon et al. [19] suggest that there is a need for changes when measuring consumption and more reliable ways of reporting, as all reviewed studies relied on self-reporting which can lead to “conservative estimates of consumption” [18].

Another limitation of the papers included in this review is that the interventions appear to address different levels of drinking in individuals. Five of the included interventions were carried out on “at risk” or “hazardous” drinkers, whereas two focused on individuals who consumed alcohol. This could lead to disparity in the results, as it is not determinable which elements of the interventions work or for whom.

Although a positive effect was found with most of the interventions in this review, the estimate of the effect could be even higher using other measures such as reporting from family members, clinicians or research assistants on physical aspects of alcohol use, or could use a “graduated frequency” approach, which begins questioning with high levels of alcohol consumption to avoid socially desirable responding [33]. This approach should be used with caution however, as starting with higher levels risks normalising them to participants and potentially encourage higher consumption [33].

All but one of the studies included in this review were carried out in the USA, with one study conducted in Demark. Further research needs to be carried out cross-culturally as there could be some non-generalisable difference. One such difference could be the healthcare system that exists in the USA: where individuals may have to pay for treatment so may be less inclined to access support. It should also be noted that there is an underrepresentation of non-white, non-educated individuals in this field.

Some of the papers included in this review address randomisation and blinding, there was frequently only limited information presented on the techniques used. This is a limitation of the papers included and should be addressed in future work. The reliability and validity of interventions have been discussed within the original papers, however they could be demonstrated better and this could be done through better intervention description and there are tools available to assist with this. The Medical Research Council [34] offers a framework for the development and evaluation of complex interventions and we recommend that this is followed in future work. The TIDieR (Template for Intervention Description and Replication) checklist and guide would also be a useful tool in intervention description and would allow authors to provide a concise description of administered interventions and where this information can be found within the individual papers.

Some papers were excluded from this review due to their inclusion of comorbidities. However, such comorbidities may be integral when investigating excessive alcohol use in older people, for example, in their study examining inpatient treatment for alcohol dependence in a group of older people, Blow et al. [35] reported that 31% of their sample were experiencing at least one psychiatric illness. Alcohol misuse and dependence can contribute to psychiatric illness and lower levels of functioning, a risk significantly higher in older adults [5, 6]. Future work should explore how different groups may respond differently to interventions or have different motivations for drinking such as using alcohol as a coping mechanism [9].

There are several strengths to this review. This review is the first study that has conducted a systematic approach to data collection focusing on interventions aimed at reducing alcohol use in older people. Previous studies have been less specific and focus on substance abuse as a whole, or have included pharmaceutical interventions which may lead to a more reductionist approach to decreasing alcohol consumption in this field, rather than the current study which focusses solely on behaviour change interventions.

This study has numerous implications for public health. Individuals are living longer and their health is important if they are to continue to live independently and enjoy later life. The development and use of interventions to reduce alcohol use in older individuals will lead to prevention, or delay of diseases such as stroke, heart disease or cancer [36].

By using the information from this review, further work should investigate what works and for whom. Targeting interventions through public health practice, could lead to a significant reduction to the health and economic burdens of excessive alcohol use. In the UK, reductions in funding across the social care sector, are affecting people in later life [37]. By introducing and utilizing cost effective behaviour change interventions, older individuals will live longer, whilst also reducing the current financial burden on the economy.

## Conclusion

This study has shown that while there is a growing evidence base for interventions for alcohol use in older individuals, there is still a need to conduct more research in the field to understand more about alcohol use in later life, and specifically understand which interventions work and for whom. Currently, interventions are aimed at general populations rather than focusing on older people. Older people are affected disproportionately by lifestyle changes such as bereavement, social isolation and loneliness and worklessness which may affect alcohol consumption [9]. More work is needed to establish the relationship between these factors and patterns of drinking in older people and also to look at varying levels of alcohol consumption across the life course, playing closer attention to stages of old age and factors such as retirement.

## Abbreviations

AUDIT: Alcohol Use Disorder Identification Tool
BA: Brief Advice
BI: Brief Intervention
CARET: Comorbidity Alcohol Risk Evaluation Tool
CARPS: Computerised Alcohol Related Problems Survey
CASP: Critical Appraisal Skills Programme
GDS: Geriatric Depression Scale
HRQL: Health Related Quality of Life
HSS: Heath Screening Survey
MCS: Mental Component Scores
ME: Motivational Enhancement
PCS: Physical Component Scores
PICO: Population, Intervention, Comparison and Outcome
PRISMA: Preferred Reporting Items for Systematic Reviews and Meta-Analyses
SHARE: Senior Health and Alcohol Risk Education
TIDieR: Template for Intervention Description and Replication
TLFB: Time Line Follow Back
